# Dr. Forbes and Homœopathy

**Published:** 1846-08

**Authors:** 


### DR. FORBES AND HOMŒOPATHY.

In a late number of our Journal we noticed an extraordinary publication by Dr. Forbes on the subject of homœopathy. This publication has drawn down upon the author a good deal of animadversion and complaint, and he has felt it necessary to enter into a long explanation in the *British and Foreign Medical Review*, of which he is editor, and in which his singular paper first appeared. We have not room, at present, to give up to any detailed account of his reply to the various letters addressed to him, but our readers, we are sure, will be gratified to see such language as the following put forth by him:

"But it never was imagined, much less contended for by the author of the article, that the treatment of diseases should or could be exclusively left, in all cases, or even in any considerable proportion of cases, to this hygienic or non-medicinal system of management; or that we are not possessed of many drugs of the noblest powers, and of known and unquestioned efficacy in the mitigation and cure of many diseases. To do this would be, in the first place, wilfully to reject evidence of the most positive kind; and, in the second place, wilfully to encounter disease with only one-half our stock of arms and ammunition. And it would seem hardly less irrational for a physician of any experience to deny the efficacy of such general means as venesection, emetics, purgatives, or the individual power of such drugs as opium, mercury, iodine, quinine, iron, colchicum, &c., in controlling diseases, than for the soldier to denounce as useless the very weapons which had often enabled him to vanquish and overcome. Here, as in the former case, the writer's anathemas were directed against the abuse or improper use of the medicines, not against their proper use. In making this explanation, however, he must still avow his belief that of a very large proportion of the drugs in common use we are entirely ignorant of the action, or whether they have any action or not; that many are certainly useless and others injurious; that of the number which possess undoubted powers in modifying the condition of the animal body, in health and disease, we have yet to learn the exact mode of action, and the amount of remedial efficacy; and that in the administration of our very best medicaments we are absurdly profuse, and thus, not seldom, convert into an evil what nature intended for, and science was capable of applying as a blessing."

